# Visuomotor learning by passive motor experience

**DOI:** 10.3389/fnhum.2015.00279

**Published:** 2015-05-15

**Authors:** Takashi Sakamoto, Toshiyuki Kondo

**Affiliations:** Department of Computer and Information Sciences, Graduate School of Engineering, Tokyo University of Agriculture and TechnologyTokyo, Japan

**Keywords:** interference, motor learning, passive movement, robot, visuomotor learning

## Abstract

Humans can adapt to unfamiliar dynamic and/or kinematic transformations through the active motor experience. Recent studies of neurorehabilitation using robots or brain-computer interface (BCI) technology suggest that passive motor experience would play a measurable role in motor recovery, however our knowledge of passive motor learning is limited. To clarify the effects of passive motor experience on human motor learning, we performed arm reaching experiments guided by a robotic manipulandum. The results showed that the passive motor experience had an anterograde transfer effect on the subsequent motor execution, whereas no retrograde interference was confirmed in the ABA paradigm experiment. This suggests that the passive experience of the error between visual and proprioceptive sensations leads to the limited but actual compensation of behavior, although it is fragile and cannot be consolidated as a persistent motor memory.

## 1. Introduction

Previous studies of human motor learning have shown that we can adapt to unfamiliar environments with dynamic and/or kinematic transformations through the active motor experience. Active motor learning has been widely investigated based on various motor tasks, such as mirror drawing (Adams, 1987; Basteris et al., 2012), shift prism (Luauté et al., 2009), visuomotor rotation (Krakauer et al., 1999; Imamizu et al., 2000; Caithness et al., 2004; Kondo and Kobayashi, 2007; Saijo and Gomi, 2010), and virtual force fields (Shadmehr and Brashers-Krug, 1997; Tong et al., 2002; Caithness et al., 2004; Bays et al., 2005; Ito et al., 2007). These studies showed that the central nervous systems (CNS) generates internal models; forward models predict future states according to the current state and action, whereas inverse models calculate an appropriate motor command based on a desired motor plan (Wolpert et al., 1995; Kawato, 1999), thereby facilitating fast and accurate movements via active interactions with the environment.

In these studies, transfer or interference of the internal models were examined because the efficient acquisition of motor skills is of general interest for human movement science research. In particular, the consecutive learning of mutually conflicting motor tasks (A and B) is known to be difficult because of retrograde interference, i.e., the motor skill required for the first task (A) cannot be retained as an internal model after 24-h rest period due to interference from a secondary task (B) experienced immediately after the first motor learning session (Brashers-Krug et al., 1996; Shadmehr and Brashers-Krug, 1997; Krakauer et al., 1999; Tong et al., 2002; Bays et al., 2005). The methodology employed in these studies is referred to as the ABA paradigm. Using the paradigm, we can investigate how a motor experience is consolidated as an internal model in our brain.

These studies demonstrate that the adjustment of feedforward motor commands is based mainly on the error between re-afferent sensory feedback and the prediction of the forward model; thus, active motor process is considered to be indispensable for motor learning. However, recent studies on robot-assisted motor experience suggest that robotic intervention facilitates the acquisition of novel motor skills (Reinkensmeyer and Patton, 2009; Bara and Gentaz, 2011; Basteris et al., 2012; Beets et al., 2012) and might also improve the motor function of hemiparesis patients (Aisen et al., 1997; Krebs et al., 1998; Riener et al., 2005; Kahn et al., 2006; Vergaro et al., 2010). In addition, brain-computer interface (BCI) based neurorehabilitation research has hypothesized that passive motor experience via a robotic exoskeleton or a functional electrical stimulation (FES) would play a measurable role in motor recovery if it is coupled to a voluntary motor intention (Takahashi et al., 2012). These studies indicate that even a passive sensorimotor experience might be effective in improving motor skills; however, our knowledge of motor learning through the passive motor experience is still insufficient compared with the active one.

To clarify the effect of passive motor experience on human visuomotor learning, we performed two motor learning experiments that comprised arm reaching tasks during visuomotor rotations guided by a robotic manipulandum. The first experiment evaluated the anterograde effect of passive motor experience on successive active motor learning. The second experiment used an ABA paradigm to investigate both anterograde and retrograde interference via passive motor experience.

## 2. Methods

### 2.1. Experimental system

**Figure 1:**
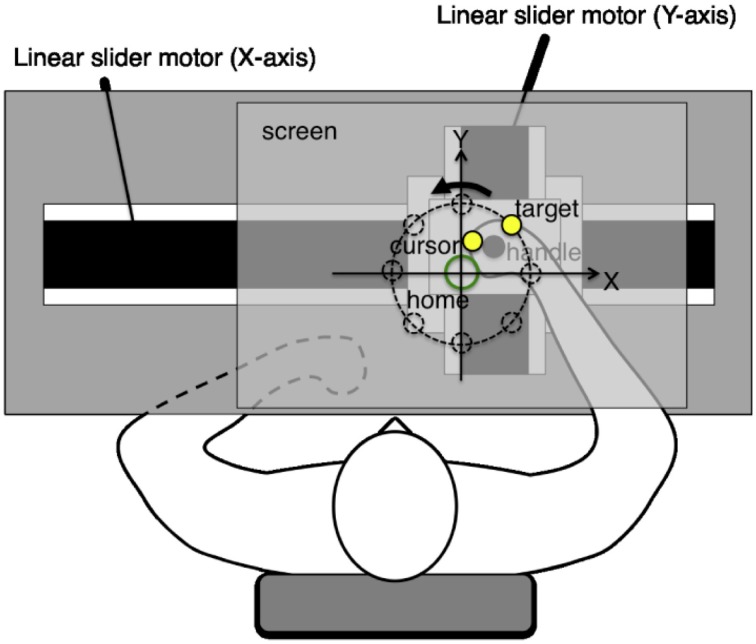
**Experimental system**. The planar robotic manipulandum comprised two orthogonal direct drive linear slider motors, which were designed to generate arbitrary virtual force fields on the handle. Subjects grasped the handle using their right hand, and they were instructed to perform reaching movements toward a target as quickly and as straight as possible. Their hands and shoulders were maintained in a horizontal plane parallel to the screen. Their elbows were also supported in the same plane by a passive arm-bracing apparatus. The horizontal screen was placed above the subject's arm, thereby preventing them from seeing their right arm movements directly.

The experiments employed a planar robotic manipulandum, as shown in Figure 1. The subjects were seated in a high back chair and they were asked to grasp the handle of the manipulandum with their right hand. During the experiments, their hand and shoulder were maintained in a horizontal plane, which was parallel to the screen, and their elbow was also kept in the plane by a passive arm-bracing apparatus. The horizontal screen was placed above the arm of the subjects, thereby preventing them from viewing their right arm movements directly. In all the experiments, a cursor, target, and home position were projected onto the screen using a ceiling LCD projector. The cursor and target were displayed as yellow solid circles (1 cm in diameter), whereas the home position was shown as an open green circle (2 cm in diameter) on the black background. These visual stimuli were always visible during arm reaching movements. The actual hand positions were recorded at 200 Hz by the robotic manipulandum.

### 2.2. Task

In the experiments, arm reaching movements under a visuomotor rotation were used as the motor learning task. The subjects were instructed to operate a handle to move the cursor toward a target as fast as and as straight as possible. The targets comprised eight candidate positions placed at 45-degree intervals on the circumference of a circle with a 10-cm radius, which was centered at the home position, where the targets appeared at one of the candidate positions in a counterclockwise sequence. In this study, we defined a reaching movement toward a target as a “trial” and a set of eight successive trials comprised a “cycle.”

Before each trial, the subjects were asked to keep the cursor within the home position circle. After 1 s, a target appeared with a beep sound, and the subjects were able to start their arm reaching movement. The trial was over if the cursor reached the target successfully, or a predetermined time limit (1000 ms) elapsed. We discarded the trials where the subjects did not leave the home position within 500 ms, thereby eliminating the trials that might have involved online cognitive processes. These were recorded as failed trials. When a reaching trial was terminated, both the cursor and target disappeared from the screen and the subject's hand was automatically returned to the home position by the robotic manipulandum.

### 2.3. Subjects

Thirty-two healthy young subjects (3 females; mean age: 22.6 ± 3.8 years) participated in the following experiments and they gave their written informed consent. Twelve subjects (3 females; mean age: 23.2 ± 4.1 years) participated in Experiment 1, and 20 different subjects (mean age: 22.3 ± 3.7 years) participated in Experiment 2. All of the subjects were right-handed, and they had no clinical history of neurological disorders, according to their self-assessment. None of the participants had any knowledge of the Experimental system, the motor learning task used in the experiments, or the purpose of the experiments. The study was approved by the ethical committee at the Tokyo University of Agriculture and Technology.

### 2.4. Experiment 1

This experiment aimed to evaluate the anterograde effect of passive motor experience on visuomotor learning. Thus, we designed two experimental groups; Group *N*1 and *P*1, i.e., “No learning” and “Passive learning” conditions, respectively. In this study, the term “passive” indicates that the subjects in the group experienced the motor learning task passively via the robotic manipulandum.

As shown in Table 1, each subject experienced a couple of experimental phases according to their group. To familiarize them with the task procedure and the operation of the robotic manipulandum, all subjects were asked to execute 5 cycles of the active reaching movement without visuomotor rotation (Null task and Practice phase). After the practice, the subjects in Group *P*1 performed 30 cycles of counterclockwise 30° visual distortion task (CCW30; TaskA) passively (Learning phase), whereas the subjects in Group *N*1 did nothing during this phase. In the passive motor experience condition, the visual cursor was moved toward the target in a straight and minimum-jerk trajectory (Flash and Hogan, 1985); thus, the subject's right hand was automatically moved by the robotic manipulandum with CW30 rotation. The velocity profile of the passive motor experience (i.e., the reference trajectory of the robot) was determined by referring to the active reaching movements during the Practice phase. During the passive movements, subjects were asked to relax and exert as little resistance force as possible. After a short break (5 min), all subjects were asked to perform TaskA actively to measure the anterograde effect of passive motor experience (Test phase, 5 cycles).

**Table 1:** **Experimental phases and groups in Experiment 1**.

**Group (Number of subjects)**			***N*1 (6)**	***P*1 (6)**
**Phase**	**Number of cycles**	**Task**	**Conditions**	
Practice	5	Null	Active	Active
Learning	30	TaskA	**No learning**	**Passive learning**
Test (5 min later)	5	TaskA	Active	Active

It may be considered that passive motor experience has a positive anterograde effect on the successive active motor learning of a visuomotor rotation task if the initial performance in the Test phase of the passive motor experience group (Group *P*1) is superior to that of Group *N*1.

### 2.5. Experiment 2

The purpose of this experiment was to examine whether the passive motor experience had a similar retrograde interference effect to active motor learning. Thus, we employed the ABA paradigm and we designed three distinct experimental groups: Group *N*2 represents “No interference,” whereas Group *M*2 and *P*2 indicate “active Movement” and “Passive movement” interference, respectively.

As summarized in Table 2, each subject experienced different experimental phases, according to their group. Similar to Experiment 1, all of the subjects first experienced 5 cycles of the Null task (Practice phase), before they were asked to execute 30 cycles of the visuomotor rotation task (CCW30; TaskA) actively in the Learning phase. Soon after the active motor learning phase (< 5 min), the subjects in the active or passive movement interference groups (i.e., Group *M*2 or *P*2) performed 30 cycles of clockwise 30° visuomotor rotation task (CW30; TaskB) under their specific experimental conditions (Interference phase), whereas the subjects in Group *N*2 did not perform an interference task. The desired velocity of the passive interference group was determined to be the same that in Experiment 1. After a 24-h rest period, all subjects were asked to perform TaskA actively to assess their retention (Test phase, 5 cycles).

**Table 2:** **Experimental phases and groups in Experiment 2**.

**Group (Number of subjects)**			***N*2 (6)**	***M*2 (8)**	***P*2 (6)**
**Phase**	**Number of cycles**	**Task**	**Conditions**		
Practice	5	Null	Active	Active	Active
Learning	30	TaskA	Active	Active	Active
Interference	30	TaskB	**No interference**	**active Movement**	**Passive movement**
Test (>24-h)	5	TaskA	Active	Active	Active

During the passive motor interference, Group *P*2 experienced CCW30 rotated proprioception with no visual shift and voluntary motor commands. It is considered that passive motor experience is sufficient to obtain an internal model to generate feedforward motor commands if the retrograde interference is confirmed in the experiment (i.e., the motor skill for TaskA is overridden by TaskB).

### 2.6. Analysis

As a performance index to evaluate the adaptation to the visuomotor rotation task, we used angular error, which we defined as the angular difference between the target and cursor vectors that originated from the home position when the hand attained the peak tangential velocity. According to our pilot study, the peak velocity was attained within 500 ms after the target's appearance, so angular error can be treated as an evaluation criterion of motor learning (i.e., the acquisition of an internal model for feedforward motor commands). To eliminate the effect of directional bias at baseline, angular errors during the visuomotor rotation task were corrected by subtracting the mean angular error during the last 2 cycles in the Practice phase for each subject (Ghilardi et al., 1995). In addition, mean angular errors across the eight successive trials (i.e., 1-cycle) were calculated for each subject.

In Experiment 1, to evaluate the anterograde transfer effect via the passive motor experience, we statistically compared the angular errors measured in the early stage of the Test phase between the groups. We hypothesized that the passive motor experience would have a positive anterograde transfer effect on the subsequent active motor learning of the identical task, we performed a one-tailed *t*-test between Group *N*1 and *P*1.

In Experiment 2, to assess the retention of learning (TaskA) against interference task (TaskB), we focused on the difference between the tail of the Learning phase and the early stage of the Test phase. Thus, we statistically compared the angular errors across groups and the focused phases using a two-way repeated-measures ANOVA and multiple comparisons test (Tukey-Kramer method).

## 3. Results

### 3.1. Experiment 1

In Experiment 1, we evaluated the anterograde transfer effect of a preceding passive motor experience on successive active motor learning. In the experiment, the total number of discarded trials was 136 [Group *N*1: 87 (480), Group *P*1: 49 (480); where the number in parentheses shows the total number of trials in each condition]. The desired peak tangential velocity for the passive interference group (Group *P*1) was set to 0.51 m/s, according to the mean peak velocities in the Practice phase.

**Figure 2:**
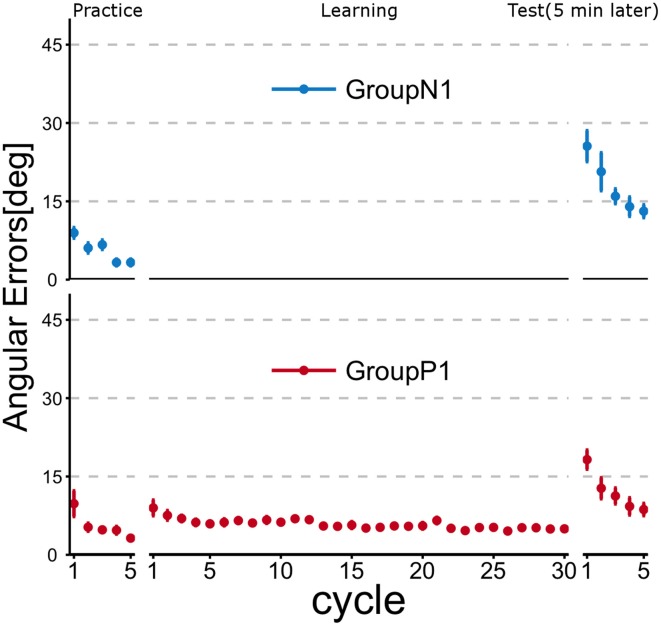
**Time course of angular errors (i.e., angular differences between the cursor and target) in Experiment 1**. Each point represents the mean angular error for each cycle across the subjects within a group. Error bars indicate ± 1 SE. In the Practice phase, all of the subjects first experienced a familiarization task (no rotation). After the Practice phase, the subjects in Group *P*1 experienced a visuomotor rotation task (TaskA, 30 degree CCW rotation) passively (i.e., by robotic manipulandum) in the Learning phase, whereas the subjects in Group *N*1 did nothing during this phase. After a 5 min rest period, all subjects were asked to perform TaskA actively to validate the anterograde effect of the passive motor experience (Test phase).

Figure 2 illustrates the time course of the mean angular errors throughout the experiment. Each point represents the mean angular error for each cycle across the subjects within a group, and the error bars indicate ± 1 SE. In the figure, Group *P*1 experienced passive arm movement during the Learning phase, and they demonstrated a relatively small angular error in the first cycle of the Test phase, but both groups exhibited obvious improvements after active motor learning even in the second cycle.

**Figure 3:**
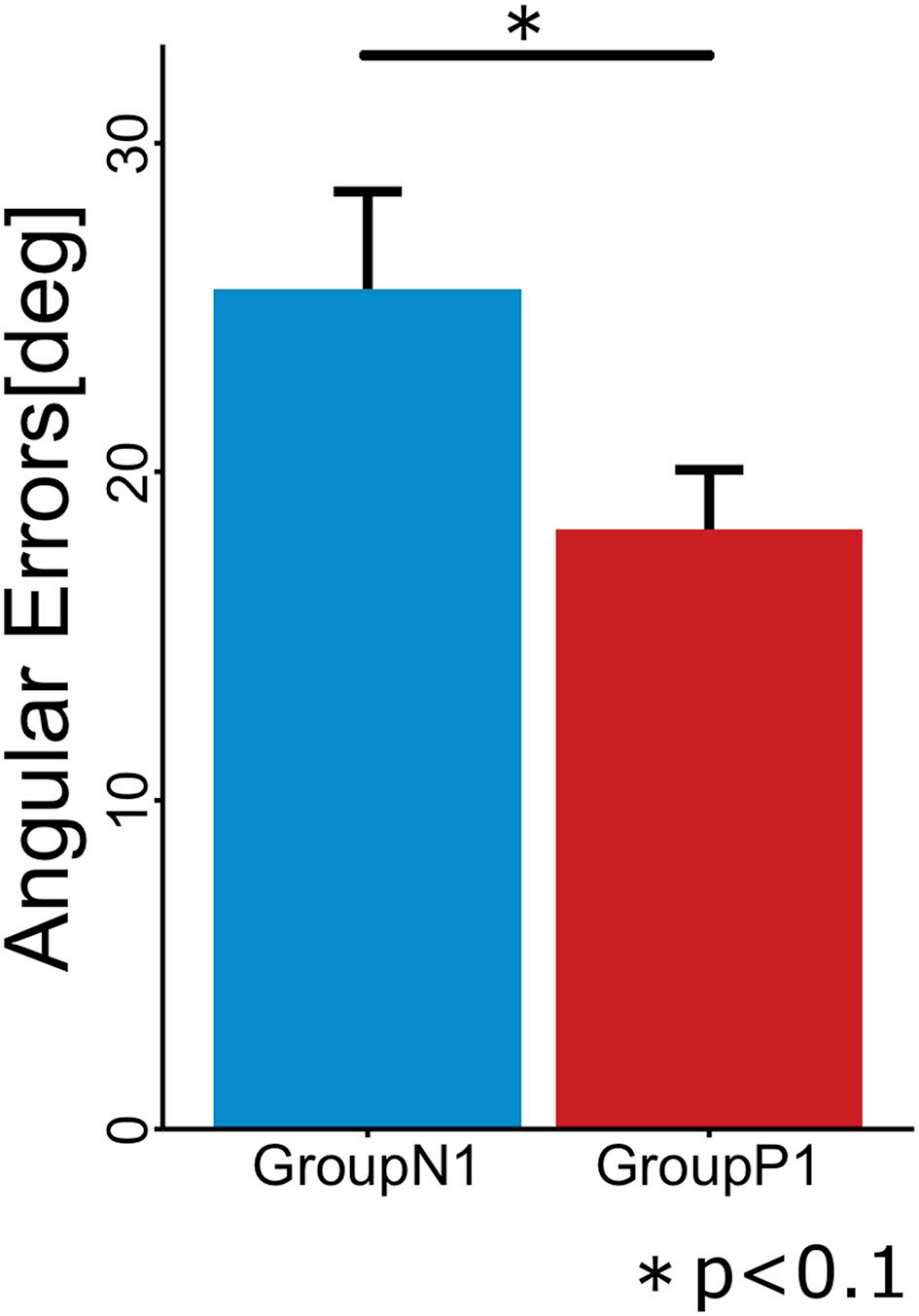
**Mean angular errors of subjects in each group during the first cycle in the Test phase**. Error bars indicate ± 1 SE.

To evaluate the anterograde transfer effect of the passive motor experience group, the angular errors measured in the first cycle of the Test phase were statistically compared (Figure 3). The *t*-test showed that there was a significant difference between the groups [*t*_(10)_ = 2.106; *p* = 0.037].

### 3.2. Experiment 2

In Experiment 2, we investigated whether the passive motor experience in TaskB interfered with the retention of the skill previously experienced in TaskA. The total number of discarded trials measured in the experiment was 408 [Group *N*2: 128 (1920), Group *M*2: 244 (4480), Group *P*2: 36 (1920)].

**Figure 4:**
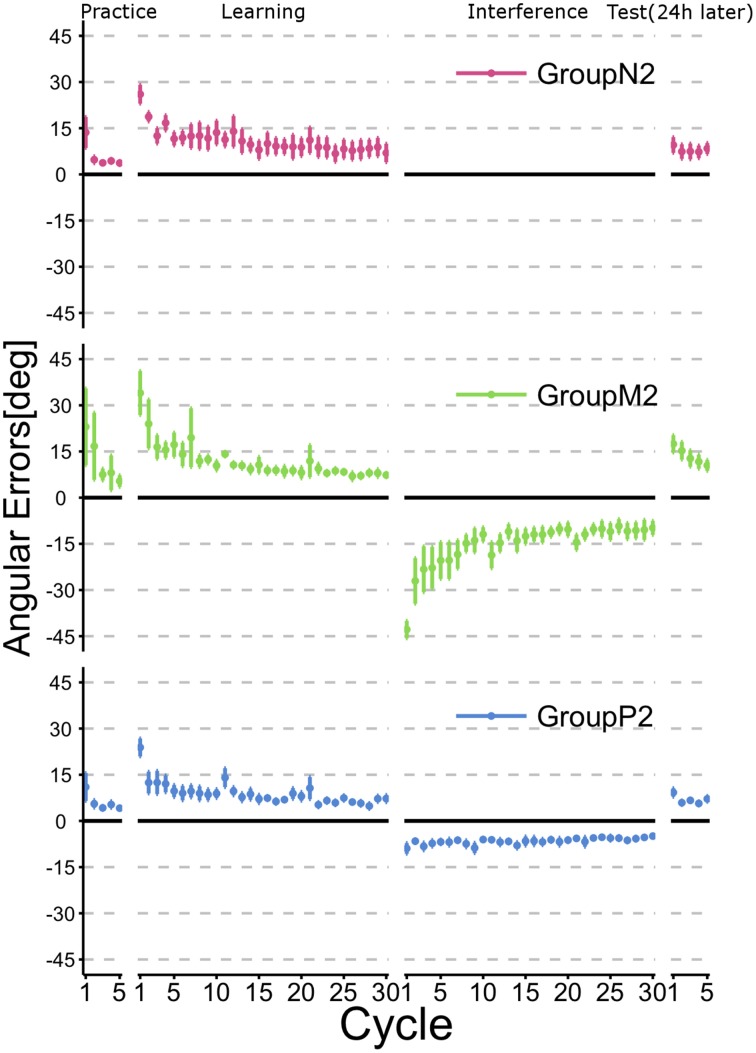
**Time course of the angular errors in Experiment 2**. Each point represents the mean angular error for each cycle across the subjects within a group. Error bars indicate ± 1 SE. First, all subjects experienced a familiarization task (no rotation) in the Practice phase, before executing a visuomotor rotation task (TaskA, 30 degree CCW rotation) actively in the Learning phase. Immediately after the active motor learning phase, the subjects in Groups *M*2 and *P*2 performed an interference task (TaskB, 30 degree CW rotation) in their specific experimental condition (Interference phase), whereas the subjects in Group *N*2 did not perform an interference task. After a 24-h rest period, all subjects were asked to perform TaskA actively to verify their retention of the skill (Test phase).

Figure 4 illustrates the time course of the mean angular errors throughout the experiment. As shown in Figure 2, each point represents the mean angular error for each cycle across subjects within a group and the error bars indicate ± 1 SE. The figure confirms that the subjects in all groups learned TaskA (i.e., CCW 30° visuomotor rotation) at the end of the Learning phase (i.e., after 30 cycles of active training). Moreover, subjects in Group *N*2, who had no interference experience in TaskB exhibited the obvious retention of TaskA before and after the Interference phase. Group *P*2 experienced TaskB passively and they also showed a similar tendency to Group *N*2. By contrast, Group *M*2 showed explicit degradation of their performance at the beginning of the Test phase.

**Figure 5:**
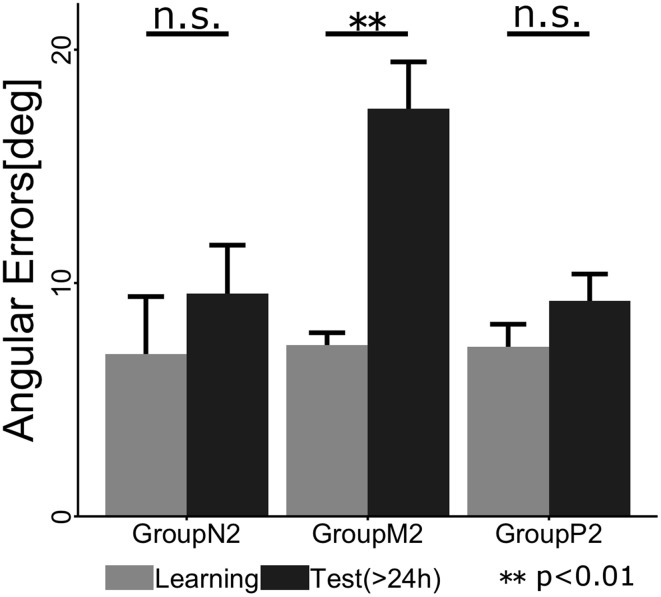
**Mean angular errors of the subjects in each group during the last cycle of the Learning phase (gray) and the first cycle of the Test phase (black)**. The error bars indicate ± 1 SE. The asterisk denotes a significant difference (*p* < 0.01) and ”n.s.” indicates no significant difference (*p* > 0.1).

To confirm whether retrograde interference occurred due to TaskB (i.e., the inverse model of TaskA was not memorized), we statistically compared the angular errors across phases (i.e., the last two cycles in the Learning phase and the first two cycles in the Test phase) and groups (Figure 5). A 2 × 3 ANOVA demonstrated the significant main effects of the phase [*F*_(1, 34)_ = 9.016; *p* = 0.00499] and group [*F*_(2, 34)_ = 5.565; *p* = 0.00811]. In addition, we confirmed that there was a significant interaction effect between the phase and group [*F*_(2, 34)_ = 5.219; *p* = 0.00106]. A *post-hoc* multiple comparisons using the Tukey-Kramer method revealed that Group *M*2 showed significant degradation of angular error before and after Interference phase (*p* <0.01), whereas Group *N*2 (*p* >0.1) and *P*_2_ (*p* > 0.1) exhibited no retrograde interference effect.

## 4. Discussion

To clarify the effect of passive motor experience on human visuomotor learning, we performed two motor learning experiments guided by a robotic manipulandum.

In Experiment 1, we hypothesized that the passive experience of arm reaching movements during visuomotor rotation (TaskA) would have a positive anterograde transfer effect on the subsequent active reaching movements. We confirmed that the mean angular error in the passive motor experience group (*P*1) during the first cycle of the Test phase was significantly lower (*p* < 0.05) than that in the no experience group (*N*1). This indicates that our hypothesis was supported, i.e., the passive motor experience contributes to the adaptation of feedforward motor commands. This result is consistent with those obtained in previous studies (Cressman and Henriques, 2010; Diedrichsen et al., 2010; Sakamoto and Kondo, 2012).

In Experiment 2, we investigated whether the passive motor experience of an interference task (i.e., TaskB) could override the motor working memory maintaining the motor skill of TaskA, i.e., retrograde interference, which was observed in the case of active motor interference. According to the results of the ABA paradigm experiment, we found that the active interference group (*M*2) showed significant degradation of angular errors during early stage of the Test phase compared with the end of the Learning phase (*p* < 0.01). This indicates that the subjects in this group suffered from retrograde interference from TaskB and thus failed to consolidate the motor skill required for TaskA, which was acquired in the Learning phase. This result is consistent with previous motor interference studies (Brashers-Krug et al., 1996; Krakauer et al., 1999; Caithness et al., 2004; Miall et al., 2004; Krakauer et al., 2005). By contrast, the passive interference group (*P*2) exhibited no significant difference before and after the Interference phase (*p* > 0.1) as observed in the no interference group (*N*2) (*p* > 0.1). Thus, it is considered that the passive motor experience via a robotic manipulandum did not interfere with the retention of the motor skill acquired in the preceding active motor learning.

The results obtained in Experiment 1 suggest that passive motor experience has a small but positive anterograde transfer effect on human visuomotor learning, whereas the results of Experiment 2 indicate that it has no interference effect on the motor memory consolidation. Therefore, these findings appear to contradict each other. Indeed, we found that the subjects who experienced active interference in Experiment 2 (i.e., Group *M*2) exhibited no anterograde interference effect after a 24-h rest period (i.e., the absolute value of the angular error in the first cycle of the Test phase was slightly smaller than that expected), although they demonstrated a distinct interference effect at the beginning of the Interference phase. Thus, it is considered that the anterograde interference effect of passive motor experience might be limited to a short time period.

A possible explanation that is consistent with our experimental findings is that passive motor experience leads to the marginal but actual compensation of behavior (i.e., adaptation of feedforward motor commands), but it is fragile and might not be consolidated as a permanent internal representation. One of the factors that may cause short-term motor adaptation is the recalibration of the visual and proprioceptive map, because Experiment 2 demonstrated that passive motor experience did not interfere with the motor working memory. In the passive motor experience conditions employed in our experiments, the subjects could not obtain any visual errors (i.e., the cursor moved straight to the target), but they might have updated their maps according to the passive proprioceptive sensation related to their hand position. Using the volatile but immediately adaptable sensory map, our brain might plan a desired trajectory and generate appropriate motor commands. Cressman and Henriques (2010) reported that motor adaptation can arise after exposure to a visuomotor distortion in the absence of movement-related errors, and even more impressively, the subjects recalibrated their sense of hand position. Diedrichsen et al. (2010) also demonstrated that the repetitive experience of a proprioceptive sensation caused similar motor adaptation (use-dependent learning). Therefore, active motor processing is not necessary for this type of motor adaptation.

In contrast, it has been demonstrated that an active motor process is indispensable for consolidating the motor memory as an internal model. The active motor process can be decomposed into several subprocesses, e.g., motor intention, motor planning, generation of motor commands, muscle contraction, and reafferent sensory feedback, however the main factor required for consolidation has not yet been clarified. Previous studies of robot-assisted motor rehabilitation suggest that inducing motor attempts in patients and providing the minimal required assistive force are beneficial for motor recovery (Kahn et al., 2006; Vergaro et al., 2010). Recent BCI neurorehabilitation studies suggest that the simultaneous experience of a voluntary motor intention and passive body movements can be effective for motor recovery (Takahashi et al., 2012). Moreover, Lotze et al. (2003) compared active and passive motor learning and concluded that voluntary motor intention is crucial for improving the motor performance and reorganizing the brain motor area. These types of motor function improvements must be caused by errors related to the bodily states anticipated in the forward internal model with re-afferent sensations. In the present study, we did not instruct the subjects to have a motor intention or to use motor imagery, but instead we asked them to relax during the passive motor experience. To enhance the utility of the passive motor experience during human motor learning, further studies should investigate the specific relationship between voluntary motor intention and the formation of motor memory via passive motor experience.

## Author contributions

TS and TK supervised the study, designed the experiments, developed the experimental system, and conducted the empirical data collection and analysis. All of the authors wrote the manuscript and approved the final manuscript.

### Conflict of interest statement

The authors declare that the research was conducted in the absence of any commercial or financial relationships that could be construed as a potential conflict of interest.
